# A Treatise on Diphtheria

**Published:** 1880-12

**Authors:** 


A Treatise on Diphtheria. By A. Jacobi, M.D., Professor of Diseases of Children, in the College of Physicians and Surgeons, New York, etc. Wm. Wood & Co., New York. 1880.

In this work of two hundred and odd pages the author takes the stand that diphtheria is not caused by micrococci, only so far as the bacteria may act on the exudations of the trachæ, as the yeast plant acts on sugar, and thus produce a septic poison that may lead to the adymanic condition cheracteristic of diphtheria. This position is sustained by much eminent authority, and in discussing it the author manifests himself a decidedly anti-parasitist. Whether putrefaction causes bacteria, or bacteria causes putrefaction is a question that has been agitating the scientific mind for some time, with the advantage, apparently, with the parasitists.

In diphtheria, we are inclined to accept the parasitic theory, and our practice, founded on that theory, has been satisfactory.

The book is well worth reading, and may do much toward settling this vexed question.

Ophthalmic and Otic Memoranda. By B. St. Roosa, M.D , Prof. of Ophthalmology in the University of New York ; Prof. of Ophthalmology and Otology in the University of Vermont, etc., and Edward T. Ely, M.D., Assistant to the Chair of Ophthalmology, University of the City of New York, etc., etc. Revised addition. Wm. Wood & Co., New York. 1880.

Cutaneous and Venereal Memoranda. By Henry G. Piffard, A.M., M.D., Prof. Dermatology, University of the City of New York, etc., etc., and George Henry Fox, A.M., M.D., Surgeon to the New York Dispensary, etc., etc. Second addition. Wm. Wood & Co., New York. 1880.

